# Novel approaches such as pharmacogenetics and liquid biopsies in tumor treatment in the era of personalized therapy

**DOI:** 10.1186/1878-5085-5-S1-A33

**Published:** 2014-02-11

**Authors:** Milena Kralickova, Vaclav Liska, Pavel Pitule, Pavel Soucek

**Affiliations:** 1 Biomedical Centre, Faculty of Medicine in Plzen, Charles University in Prague, Plzen, Czech Republic

## Objectives

Current findings in the biology of tumors and also new tools for genome analysis have opened clinical perspectives in oncology, generally termed as 'personalized medicine'. Pharmacogenomics examines how the benefits and adverse effects of a drug vary among patients in a target population by analyzing genomic profiles of individual patients. Tailored treatment to a given patient presenting with a given tumor is a must in these days. Despite outstanding results in colorectal cancer, lung cancer and melanoma, only a few predictive biomarkers are currently justified in routine clinical practice. Unfortunately so far there is still a gap between the growing number of identified deregulated pathways and their actual implementation as part of clinical routine.

Our current focus is on ATP-binding cassette (ABC) transporters that contribute to development of resistance to anticancer drugs via ATP-dependent drug efflux [1,2]. The associations between the expression of ABC transporters and outcome of carcinoma patients, e.g. breast carcinoma patients, could elucidate some question in pathophysiology of the disease and contribute to new treatment strategies [3].

As long as endometriosis, characterized by the presence of endometrial tissue outside of the uterine cavity, is one of the most common gynecologic disorders in women of reproductive age and has many similarities with cancer, we focus on this disease as well. Endometriosis is an inflammatory, estrogen-dependent condition associated with pelvic pain and infertility. Decreased quality of life is the most important predictor of direct health care and total costs [4]. New interdisciplinary research proposals in the field of endometriosis research will contribute to its better understanding, earlier diagnosis and personalized treatment.

Another issue of our research is the oncofertility. As long as chemotherapeutics, radiation, and surgery can impact the future fertility of men, women, and children with cancer the fertility impairment and loss due to cancer or its treatment is a significant survivorship consideration.

## Suggested technologic approaches

Systems biology approaches are the must for an easier implementation of personalized medicine at the bedside. The new Biomedical centre of the Faculty of Medicine in Pilsen is part of the Charles University. It houses a multidisciplinary team of researchers engaged in collaborative research with partners across the globe. One of the primary focuses of the centre is the identification of predictive biomarkers of drug safety and efficacy with the aim of translation from 'bench-to-bedside'. The Centre is in a close vicinity of the University Hospital in Pilsen and emphasis the education of not only medical students but also clinicians and scientists.

## Expected impact if the project will succeed

New personalized medicine biomarkers, both prognostic and predictive, will contribute to better classification of patients into subgroups for treatment recommendation and also to new personalized treatment of cancer and endometriosis. Our aim is to support our young patients and children also from the oncofertility point of view and to save them hope for future fertility.
